# Bite‐DNA Shows Substantial Browsing on Willows (*Salix* spp.) by North American Bison in Yellowstone National Park

**DOI:** 10.1002/ece3.73354

**Published:** 2026-03-31

**Authors:** Julia L. Jansson, Robert Spitzer, Jaelle Caitlin Brealey, Göran Spong

**Affiliations:** ^1^ Molecular Ecology Group, Department of Wildlife, Fish and Environmental Studies Swedish University of Agricultural Sciences Umeå Sweden; ^2^ Land and Biodiversity Norwegian Institute for Nature Research Trondheim Norway

**Keywords:** bison, browsing, riparian, trophic ecology, ungulates, willows

## Abstract

Riparian willows (*Salix* spp.) in Yellowstone National Park have long been shaped by ungulate browsing, yet the specific contribution of individual herbivore species remains unclear. We applied a bite‐DNA metabarcoding approach, extracting saliva DNA from browsed willow twigs, to directly identify the browsing community across six northern range riparian sites. Mammalian DNA was successfully assigned for more than half of the collected bite samples, revealing browsing by moose ( *Alces alces* ), North American bison ( *Bison bison* ), elk ( *Cervus canadensis* ), deer (*Odocoileus* sp.), bighorn sheep ( *Ovis canadensis* ), and jackrabbit ( *Lepus townsendii* ). Contrary to the traditional view of bison as primarily grazers, bite‐DNA showed that bison were the most frequent browsers of willows, present at all sites and contributing the majority of browsing bites. Elk, historically considered the primary browser on riparian shrubs, were detected less often, whereas mule deer browsing was consistently recorded and frequently exceeded elk. Browsing height largely overlapped among species and was significantly higher for bighorn sheep than for bison and mule deer. Diameter of browsed twigs did not differ significantly between species. Browsing composition varied locally without clear spatial patterns, suggesting that site‐level factors shape where different ungulates browse willows. Our results demonstrate substantial bison browsing on riparian willows and highlight shifting herbivore impacts on Yellowstone's riparian ecosystems.

## Graphical abstract

Riparian willows in Yellowstone National Park are shaped by ungulate browsing, but species‐specific contributions remain unclear. Using bite‐DNA metabarcoding of browsed willow twigs across six northern range sites, we found that American bison were the most frequent browsers, exceeding elk and often mule deer. These bite‐scale results demonstrate substantial bison impacts and highlight shifting patterns of herbivory in Yellowstone's riparian ecosystems.

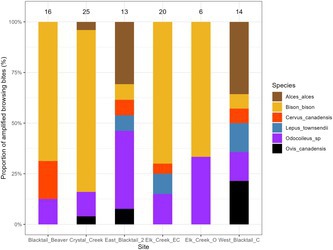

## 1 Introduction

Yellowstone National Park has long been recognized as a globally important natural laboratory for understanding how trophic interactions shape ecosystems, particularly through the interplay of large herbivores, predators, and vegetation dynamics. Decades of research have documented how the removal and subsequent restoration of apex predators have altered ungulate behavior, browsing intensity, and riparian vegetation structure (Beschta and Ripple 2008; Houston 1982; Ripple and Beschta 2004). The park's northern range, in particular, represents one of the most detailed natural experiments linking predator regulation, herbivore pressure, and vegetation recovery (Beschta and Ripple 2016; Beyer et al. 2007; Creel and Christianson 2009; Marshall et al. 2013; Painter et al. 2015).

The landscapes of Yellowstone's northern range have undergone substantial ecological change over the past century (Wagner 2006). In the early 1900s, the riparian zones of rivers were dominated by extensive willow (*Salix* spp.) communities interspersed with numerous beaver ( *Castor canadensis* ) dams that shaped stream hydrology and floodplain structure (Hobbs et al. 2024). Beaver activity elevated and stabilized water tables, creating conditions ideal for willow growth and regeneration (Larsen et al. 2021). Through dam building and soil disturbance, beavers also promoted the bare, moist substrates necessary for the establishment of willow seedlings, thereby maintaining a dynamic *beaver‐willow state* characteristic of the historical northern range (Hobbs et al. 2024; Wolf et al. 2007).

The transition from this historical beaver‐willow state to the modern *elk‐grassland state* in northern Yellowstone likely had multiple drivers (Hobbs et al. 2024). Regional climatic changes such as warming and drying following the Little Ice Age (Houston 1982; Persico and Meyer 2009) and fire suppression that reduced the creation of bare seedbeds for willow establishment (Despain 1986; Houston 1982) have been implicated. However, these broad environmental changes cannot alone explain the local collapse of riparian willows in Yellowstone, as they also affected regions outside the park where elk ( *Cervus canadensis* ) populations were kept low through regulated hunting (Hobbs et al. 2024). This suggests that the dominant cause of riparian willow decline was intense elk browsing following predator removal (National Research Council 2002; Wagner 2006). After wolves ( *Canis lupus* ) and cougars ( *Puma concolor* ) were extirpated by the 1920s, elk populations rose dramatically, aided by habitat conversion, supplemental feeding, and refuge from hunting outside the park (Houston 1982; Wagner 2006). Excessive browsing suppressed willows and aspen ( *Populus tremuloides* ), eliminating the primary food and building materials for beavers and leading to their decline (Baker et al. 2005; Chadde and Kay 1991).

Elk culling during the 1950s–1960s occurred after the transition toward elk‐dominated grasslands was already well underway (Wagner 2006), and the subsequent cessation of culling allowed elk numbers to expand again (Singer et al. 1998). The restoration of the park's large predator guild, which included the reintroduction of wolves in 1995 and the recovery of grizzly bear ( *Ursus arctos horribilis* ) and cougar populations during the 1980s–1990s (Hamlin et al. 2008; Ruth et al. 2019), reestablished partial top‐down control of elk populations. Additionally, greater access to areas outside the park with reduced intraspecific competition has led most of the northern range elk herd to winter outside Yellowstone National Park (Mosley and Mundinger 2018). However, as elk numbers declined, populations of other large herbivores, particularly North American bison (*Bison bison*, hereafter simply “bison”), increased.

Since the 2000s, bison numbers have risen sharply, surpassing elk after 2012, with total ungulate biomass and browsing intensity increasing in parallel (Hobbs et al. 2024). The traditional view of bison as primarily grazers with minimal browsing impact has been challenged in recent decades. In Yellowstone, bison browsing effects on willows have been inferred indirectly through browsing‐height analyses (Painter and Ripple 2012), experimental exclosures (Kauffman et al. 2025), and measurements of willow recovery (Painter and Tercek 2020). More directly, camera‐trap data indicate that bison now use willow habitats more frequently than elk and browse willows in about 20% of observations (Hobbs et al. 2024).

Even more recently, fecal DNA metabarcoding has enabled multi‐species analyses of resource partitioning among pronghorn ( *Antilocapra americana* ), bighorn sheep ( *Ovis canadensis* ), mule deer ( *Odocoileus hemionus* ), elk, and bison in Yellowstone (Hoff et al. 2025; Littleford‐Colquhoun et al. 2024). However, this method cannot reliably link fecal samples to precise feeding locations, and quantitative estimates of diet composition from metabarcoding remain challenging (Deagle et al. 2019; Johnson et al. 2025; Lamb et al. 2019). Consequently, the relative contributions of multiple herbivores to overall browsing pressure on riparian willows in Yellowstone remain poorly understood.

Here, we applied a ‘bite‐DNA’ approach (Jansson et al. 2025; Nichols et al. 2015), that is, extracting and sequencing mammalian DNA from saliva left on browsed willow twigs, to quantify the composition of the browsing community in northern Yellowstone. This method provides a direct and spatially explicit means of identifying which herbivore species consume riparian willows. By linking browsing bites to specific species, we aimed to accurately estimate the relative contributions of bison, elk, and other mammalian herbivores to current willow browsing pressure. This, in turn, contributes to a more refined understanding of how changing herbivore assemblages influence riparian willow dynamics in Yellowstone's northern range.

## 2 Methods

### 2.1 Study Area and Sample Collection

**FIGURE 1:**
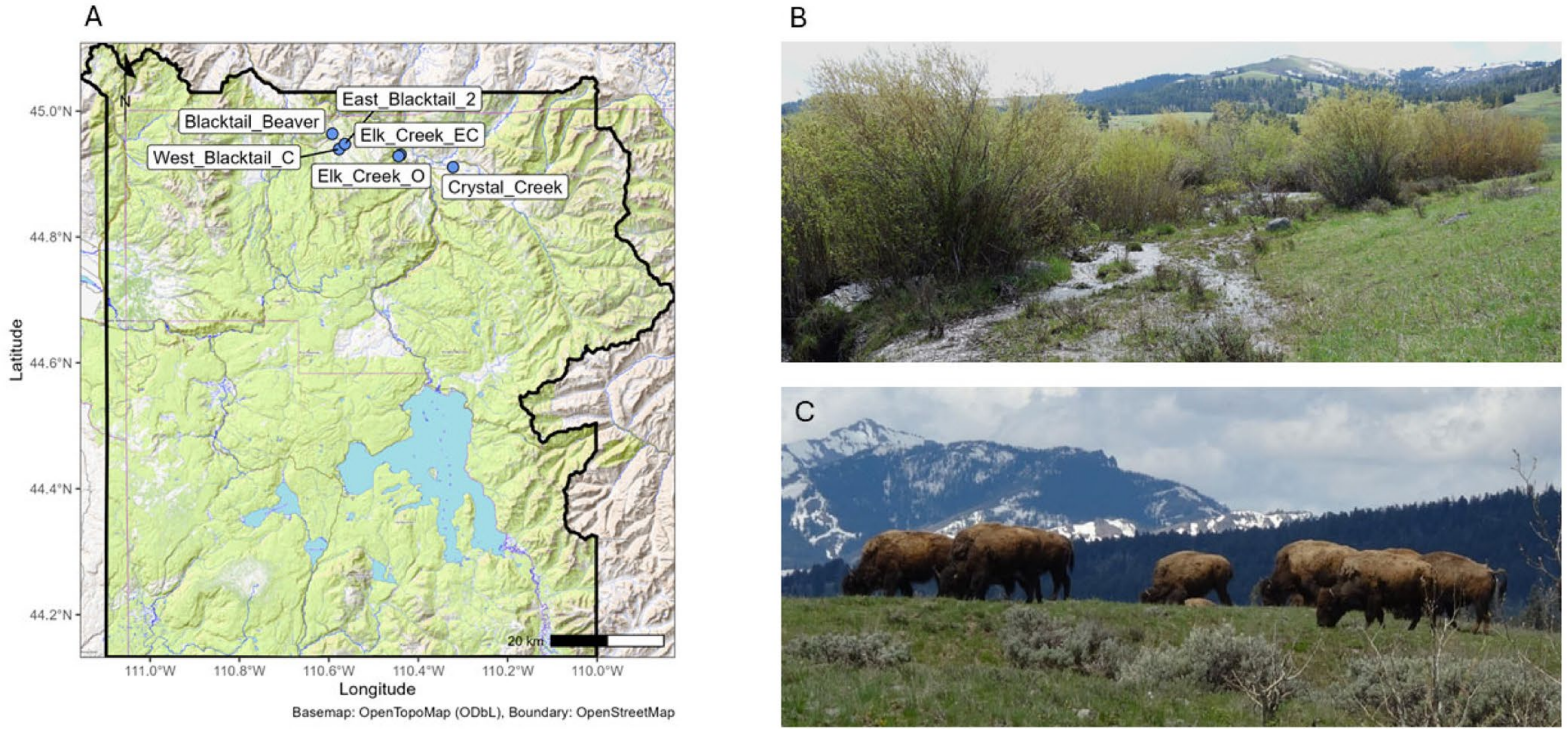
(A) Location of the six riparian study sites within northern Yellowstone National Park, USA. (B) Example of a riparian willow (*Salix* spp.) thicket at the Crystal Creek site. (C) A group of foraging North American bison in northern Yellowstone. Photos: J. L. Jansson.

Our study sites (Figure 1A) were located within the central part of Yellowstone's northern range, south of the Yellowstone River and west of the Lamar Valley. The northern range of Yellowstone National Park, USA, covers roughly 100,000 ha and serves as critical winter habitat for the park's largest elk herd (Houston 1982). Average elevation is around 2000 m, with a mean annual precipitation of about 410 mm, 44% of which falls as snow (Wolf et al. 2007). The landscape consists of rolling glacial till hills, where lower elevations are dominated by *Artemisia tridentata* and *Elymus smithii* , transitioning at higher elevations to forests of *Pseudotsuga menziesii, Picea engelmanni*, and *Pinus contorta* . The cool, semi‐arid steppe is interspersed with wetter patches and ribbons of vegetation maintained by surface and groundwater (Hobbs et al. 2024). The riparian zone of streams and small wetlands supports communities of *Carex aquatilis* and willow thickets (*Salix* spp.), often kept short by heavy browsing (Bilyeu et al. 2008).

Browsing bite samples from willow shrubs were collected in late May 2018, shortly after bud break. DNA can typically be amplified from bite marks exposed for up to 12 weeks, although PCR success declines with time (Nichols et al. 2012). Thus, the collected samples primarily reflect winter and early spring browsing activity. We did not measure snow depth at our sites, but data from nearby stations of the National Water and Climate Center (NWCC), U.S. Department of Agriculture (USDA) (https://nwcc‐apps.sc.egov.usda.gov/imap/), indicate snow depths of approximately 120 cm during February–March 2018 at two stations closest to our study area and at similar elevation (Canyon and Sylvan Road).

Six riparian willow sites were sampled (Figure 1A; Table A1 in Appendix 1), each dominated by two common willow species ( *Salix geyeriana* and *S. bebbiana* ; hereafter collectively referred to as “willows”). Shrubs were randomly selected at each site with a target of eight shrubs per site. From each shrub, approximately six browsed twigs were clipped ~15 mm below the bite mark, placed individually in paper envelopes, and stored with silica gel. Twig diameter was measured immediately below the clipping point to avoid contamination, and bite height was measured vertically from the ground. To capture the full browsing range, twigs were collected from the lowest to highest available bite marks, and only fresh bites (those not grayed with age) were sampled. Clippers were flame‐sterilized between samples to prevent DNA carryover.

Eight unbrowsed control twigs were collected across sites using the same procedure. All samples were kept at room temperature for less than 1 week during transport and subsequently stored at −20°C upon arrival at the SLU laboratory in Umeå, Sweden.

### 2.2 DNA Extraction and PCR

DNA was extracted from the twig samples using the Nucleospin Soil kit (Macherey‐Nagel), following the modified protocol described in Jansson et al. (2025), which adapts the kit's original soil‐based procedure for twig material. Briefly, the twig samples were vortexed in lysis buffer S1 together with ceramic beads, then centrifuged, after which the remaining solid plant material was removed from the extraction process.

Saliva DNA recovered from bite marks on browsed twigs is typically present in low quantities and often degraded. Short DNA fragments are therefore more likely to be successfully amplified from such samples (Deagle et al. 2006; Deiner et al. 2017). Following this rationale and previous work identifying ungulate species from browsed twigs using short diagnostic PCR fragments (74–83 bp) (Nichols and Spong 2017; Nichols et al. 2012), we chose to amplify a 108–121 bp region of the 16S mitochondrial rRNA gene. Library preparation was executed according to the protocol by Hugerth et al. (2014) with two consecutive PCR procedures. The first PCR amplified the region of interest and attached Illumina adapters to the amplicons that are used in the next step. The primers used were Mamm02, forward 5‐CGAGAAGACCCTRTGGAGCT‐3 and reverse 5‐CCGAGGTCRCCCCAACC‐3 (Giguet‐Covex et al. 2014; Taberlet et al. 2018) with Illumina adapter primers attached forward 5′‐ACACTCTTTCCCTACACGACGCTCTTCCGATCT‐[Mamm02], reverse AGACGTGTGCTCTTCCGATCT‐[Mamm02]. To optimize the PCR protocol, test amplifications were conducted and evaluated by gel electrophoresis using DNA previously extracted from tissue samples of moose, bison, elk, and mule deer, in addition to DNA extractions from twig samples. The final protocol was conducted with 17.5 μL of Kapa HiFi master mix (Kapa Biosystems, Woburn, MA, USA), 1.05 μL of each primer, 4.9 μL of template DNA, and 7 μL of nuclease‐free water. The amplification conditions consisted of a touchdown PCR of 2 min at 98°C for pre‐denaturation, followed by denaturation at 98°C for 20 s, annealing at 64°C (with 1°C decrements from 64°C to 56°C at every cycle) for 20 s, and elongation at 72°C for 15 s. The reaction was finished with a final elongation step of 2 min at 72°C. The product was then sent to the Science for Life Laboratory/NGI (Solna, Sweden) where bead purification, the second PCR, and sequencing on a MiSeq (Illumina Inc., San Diego, CA, USA) were performed according to the protocol in Hugerth et al. (2014).

### 2.3 DNA Metabarcoding

Primer sequences were trimmed from the forward and reverse reads using cutadapt v.3.7 (Martin 2011) with a minimum length of 15 bp overlap and an allowed 15% mismatch. Because of the poor quality of the reverse reads, only the forward reads were retained for analysis. Quality filtering, error correction, and amplicon sequence variant (ASV) generation were performed using the DADA2 v1.9 package in R (Callahan et al. 2016). All forward reads were truncated at the first base with a quality score < 10 and then trimmed to 92 bp to remove the presence of primer and adapter sequences in the 3′ ends. Reads with ambiguous bases, > 2 expected errors, or a length < 92 bp after trimming were removed. Error rate models with enforced monotonicity were estimated for the forward sequences and ASVs were inferred for each sample. Chimeric ASVs were assessed on a per‐sample basis, and an ASV was removed if it was flagged as chimeric in > 90% of the samples in which it occurred.

Taxonomy was assigned to each ASV using an RDP classifier from RDP Tools (Wang et al. 2007) against a custom non‐redundant database of Yellowstone mammalian reference sequences from *Bison, Cervus, Odocoileus, Alces, Ovis, Antilocapra, Lepus, Castor, Marmota*, *Sylvilagus*, *Urocitellus*, *Tamiasciurus*, *Tamias*, *Ochotona* and *Ursus* species, as well as non‐native species *Oreamnos americanus* and *Equus caballus* , and potential contaminants *Homo sapiens* , *Mus musculus* and *Bos taurus* (downloaded from Genbank on 07‐01‐2025 and 09‐03‐2026, Table A2 in Appendix 1). To check the completeness of our custom database, and for spurious assignments, we also performed a megablast search (Morgulis et al. 2008) against both the custom database and the NCBI nucleotide non‐redundant database (downloaded 21‐10‐2024, Sayers et al. (2022)). ASVs were assigned to the genus‐ or species‐level when they had > 90% confidence score at that level in RDP and a best BLAST match with > 90% identity and > 85% coverage against both the custom mammalian database and the NCBI database. ASVs not satisfying these requirements were excluded as either non‐target sequences, or sequences with ambiguous taxonomy (e.g., those that could only be assigned to the family‐ or order‐level). ASVs assigned to the genera *Homo*, *Mus*, or *Bos* were also classed as non‐target sequences. All other non‐target ASVs had either poor‐quality hits or no hits in the NCBI Genbank blast (likely sequencing artifacts) or were assigned to taxa outside the scope of this study (e.g., *Salix* and various bacterial and viral taxa; see Table A3 in Appendix 1).

The target ASVs had an average RDP genus‐level confidence score of 98.4% (custom mammalian database) and an average BLAST identity of 98.1% (custom mammalian database) and 98.9% (NCBI Genbank database). Target ASVs assigned to *Lepus, Ovis*, and *Odocoileus* could not be confidently assigned at the species‐level because of high similarity of the reference sequences among species. However, on the basis of known species occurrences in the area, all *Lepus* ASVs are likely *L. townsendii* and all *Ovis* ASVs are likely *O. canadensis* . *Odocoileus* ASVs may be *from O. hemionus* or *O. virginianus* . Finally, the most abundant target herbivore species in each sample was identified. Samples with less than 1% target herbivore sequences were classified as “not amplified” and excluded from further analysis.

### 2.4 Statistical Analysis

All statistical analyses were conducted in R version 4.4.2 (R Core Team 2024). Results were considered significant at α = 0.05. To assess whether amplification success differed among sites, we fitted a generalized linear mixed model (GLMM) using the *glmer*() function from the lme4 package (Bates et al. 2015). Amplification success was modeled as a binary response variable (0 = no amplification, 1 = amplification), with site as a fixed effect and tree ID as a random effect to account for non‐independence among samples from the same tree. Model significance was evaluated by comparing the full model to a null model (intercept only) using a likelihood ratio test (*anova*()). Model diagnostics were conducted using the *simulateResiduals*() function from the DHARMa package (Hartig 2024) to assess model fit and residual patterns. The proportion of variance explained by the model (marginal and conditional *R* ^2^) was calculated using the *r.squaredGLMM*() function from the MuMIn package (Bartoń 2010).

Similarly, we tested for differences in browsing height and the diameter of browsed twigs among browsing species using linear mixed models (LMMs) fitted with the *lmer*() function from the lme4 package, assuming a Gaussian error distribution. Species was included as a fixed effect. We initially specified a nested random‐effects structure (tree ID nested within site), but this resulted in singular fits because the among‐site variance was effectively zero (as indicated by *VarCorr*()). Consequently, only the tree ID was retained as a random effect. Overall model significance was evaluated via *anova*(), comparing the full model against a null (intercept‐only) model, as described above. Model fit and assumptions were assessed using the same diagnostic procedures outlined previously (via the DHARMa package). Post hoc pairwise comparisons among species were conducted using the emmeans package (Lenth 2017).

To assess whether browsing composition (i.e., the proportional contribution of browsing bites by each browsing species) differed among sites, we used a chi‐squared test for homogeneity (*chisq.test*()) with simulated *p*‐values on the basis of 10,000 Monte Carlo replicates to account for small sample sizes. The same approach was used for pairwise comparisons between sites with Holm‐adjustment of *p*‐values. However, not all site pairs could be compared because, in some cases, one or more browsing species were absent from both sites (i.e., the corresponding contingency table columns contained only zeros). In such cases, the chi‐squared test could not be performed because the absence of observations prevents estimation of expected frequencies and, therefore, statistical assessment of variation between those site pairs.

Finally, we examined potential spatial effects by testing for spatial autocorrelation in browser community composition. Specifically, we compared Bray‐Curtis dissimilarities in browsing composition (on the basis of the relative abundance of browsing bites per browsing species and site) with geographic distances between sites using a Mantel test.

## 3 Results

After removing control samples, a total of 173 browsing bite samples remained in the final dataset. Of these, 94 (54.3%) were successfully assigned to target herbivore species, whereas the remaining 79 (45.7%) were classified as “not amplified”. DNA metabarcoding identified six browsing species: moose ( *Alces alces* ), bison, elk, jackrabbit ( *Lepus townsendii* ), deer (*Odocoileus* sp.), and bighorn sheep. The *Odocoileus* sequences could only be resolved to genus level, but most likely represent mule deer ( *O. hemionus* ), which are abundant in the study area. White‐tailed deer ( *O. virginianus* ) are also present but occur at low densities (https://www.nps.gov/yell/learn/nature/mule‐deer.htm).

**FIGURE 2:**
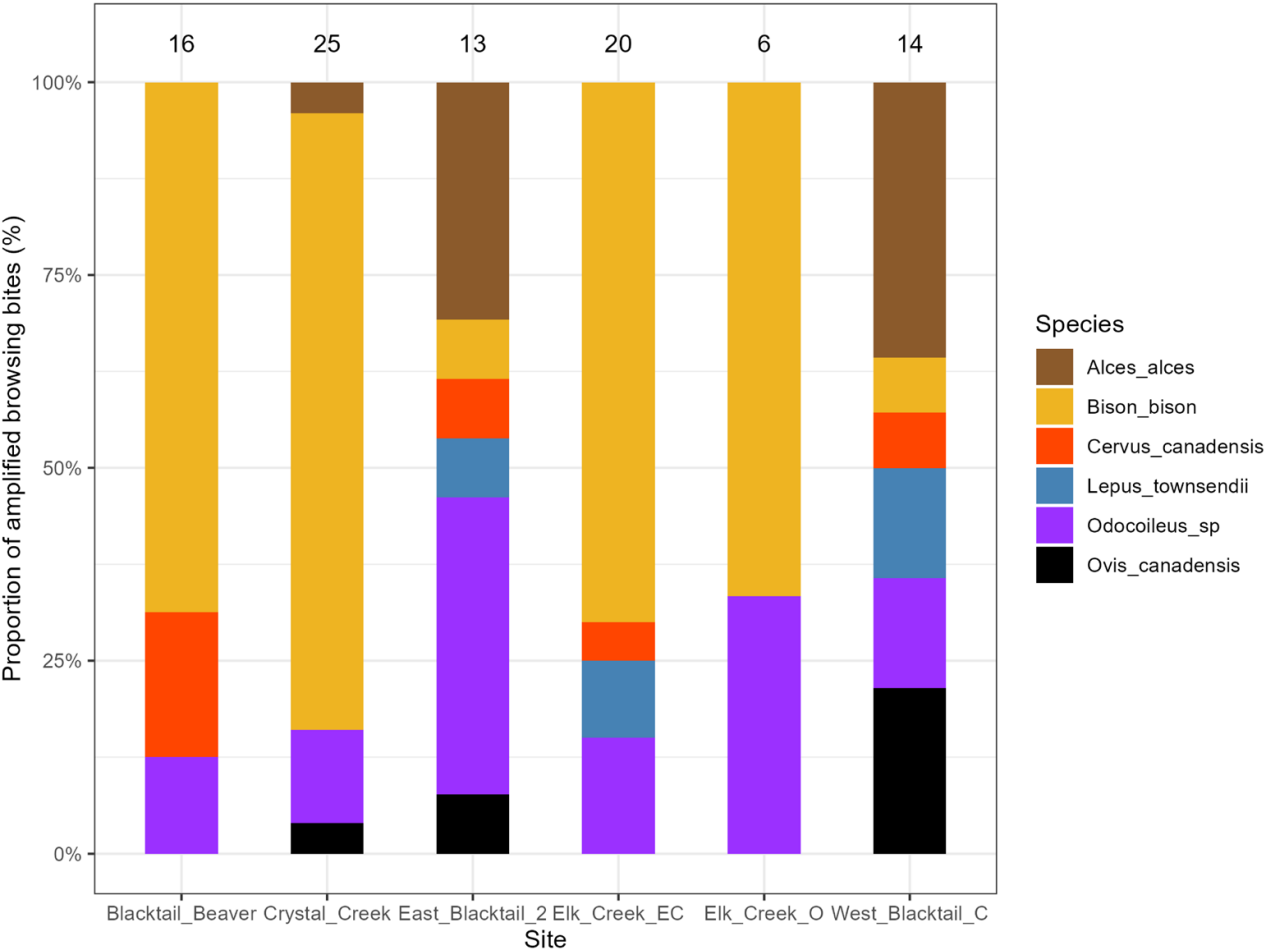
Barplot showing the composition of amplified browsing bites across six sites in Yellowstone National Park. Colors represent browsing species, and numbers above bars indicate the number of successfully assigned browsing bite samples per site.

We found no significant effect of site on amplification success (χ^2^ = 8.46, *p* = 0.13), and site explained only a small proportion of the variation (marginal *R* ^2^ = 0.08). Only bison and deer were detected at all six sites, and bison browsing accounted for the largest proportion of browsing bites at four of the six sites (Figure 2; Table A1 in Appendix 1).

Browsing composition differed significantly among sites (χ^2^ = 59.95, *p* < 0.001), with the strongest contrasts observed between the Blacktail Beaver and West Blacktail C sites and the Crystal Creek and West Blacktail C sites (Table A4 in Appendix 1). However, browsing composition did not show clear spatial patterns, as there was no significant correlation between community dissimilarity and geographic distance (Mantel *r* = 0.07, *p* = 0.47).

**FIGURE 3:**
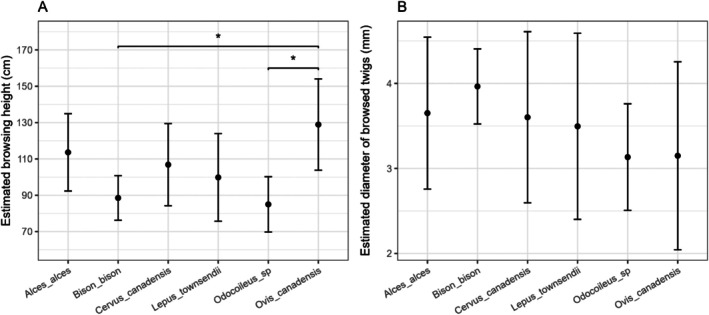
(A) Estimated browsing height and (B) diameter of browsed twigs for six browsing species in Yellowstone National Park. Points show model‐based marginal means, and error bars represent 95% confidence intervals. Significant pairwise differences are indicated by asterisks.

Browsing height, but not the diameter of browsed twigs, differed significantly among browsing species (χ^2^ = 15.28, *p* = 0.004). Post hoc pairwise comparisons showed that bighorn sheep browsed at greater heights than both bison (estimate = 40.40 ± 13.02 cm SE, t = −3.10, *p* = 0.031) and mule deer (estimate = 43.93 ± 13.92 cm SE, t = −3.16, *p* = 0.027; Figure 3).

## 4 Discussion

In line with suggestions from earlier studies (Hobbs et al. 2024; Painter and Ripple 2012), we found bison, rather than elk, to be the most prominent browser on willows in northern Yellowstone, highlighting the species' foraging flexibility. Bison browsing occurred at all six sites and accounted for 54% of all identified browsing bites. In comparison, elk browsing was detected at only four sites and represented just 6% of total browsing bites (Figure 2, Table A1 in Appendix 1). Browsing by smaller deer (*Odocoileus* sp., likely mule deer) also occurred at all sites, accounting for 18% of browsing bites, three times more than elk. These results strongly suggest that elk, although still an important member of the browsing community, may no longer be the primary driver of browsing pressure on willows in the northern range.

Despite substantial differences in body size among species, we found little evidence of vertical partitioning in browsing height. The browsing height of the smallest species, the jackrabbit, overlapped with that of the tallest, moose. Only bighorn sheep browsed significantly higher than both mule deer and bison. This limited separation in browsing height likely reflects the generally small stature of willows (typically < 2 m) and the presence of snow during late winter and early spring, which together constrained the accessible browsing strata. Consistent with Painter and Ripple (2012), most bison browsing occurred below 1 m in height. The tendency of bighorn sheep to browse higher remains unclear, but one possible explanation is heightened vigilance toward predators such as wolves and cougars. Increased perceived predation risk can cause prey species to forage at higher strata to maintain better visibility of their surroundings (Churski et al. 2021), although this hypothesis could not be tested here. Alternatively, and perhaps more likely, bighorn sheep may rear up on their hind legs to reach higher bites (pers. comm. reviewer), or their browsing may have occurred earlier in the season when snow depth was greater, although neither explanation can be evaluated with our data.

Browsing composition varied significantly among sites, but this variation did not follow geographic distance, which suggests that local habitat conditions and herbivore behavior, rather than broader spatial structuring, shape where different species browse willows. Future research should assess whether such locally distinct browsing communities translate into spatially uneven willow recovery and long‐term riparian ecosystem dynamics across the northern range.

We did not detect any browsing by pronghorn, which is consistent with previous dietary studies indicating that willows are not a preferred food item. During winter and spring, pronghorn diets in Yellowstone can contain substantial amounts of shrubby browse such as sagebrush (*Artemisia* spp.), comprising approximately 20% to 60% of their diet (Barnowe‐Meyer et al. 2017). However, the same study found willows in only 1 month (February) and at a very low proportion (0.3%). Similarly, fecal DNA metabarcoding rarely detected willow in pronghorn feces (Hoff et al. 2025).

Our results represent only winter and early spring browsing and may not be representative of other seasons. Moreover, a substantial proportion of putative browsing bites (46%) failed to amplify, for reasons that remain unclear. Species‐specific amplification biases could exist and may have influenced our estimates. In addition, because we targeted a relatively short (~108–121 bp) fragment to maximize amplification success from degraded saliva DNA on browsed twigs, taxonomic assignment using the RDP classifier may be somewhat less accurate than with longer sequences (e.g., ~250 bp) (Lan et al. 2012). However, amplification success did not differ among sites, and the consistent detection of bison and mule deer across all locations still suggests a greater prominence of these species compared to elk. We also could not link browsing bites to individual animals. For instance, a herd of bison moving through an area with many individuals taking a few bites each may have different ecological implications than one or a few individuals of another herbivore species remaining in an area and repeatedly browsing over a longer period. Thus, the relative importance of willows in a species' overall diet cannot be inferred directly from the proportion of browsing bites at a site without information on residency time and broader dietary context. Furthermore, since we sampled only willows, the composition of browsing bites does not necessarily reflect the full foraging community or the abundance of browsing animals at each site, as individuals may have fed on other available vegetation. Nevertheless, our findings clearly highlight riparian willows as an important winter and spring food resource for Yellowstone's ungulates with the exception of pronghorn and indicate that bison currently exert the strongest browsing pressure on these plant communities. This pattern also has broader implications, providing direct evidence that contemporary browsing on riparian willows in northern Yellowstone may be dominated by bison rather than elk. This shift in herbivore identity has implications for ongoing debates about trophic cascade strength in Yellowstone, including recent exchanges over how strongly predator restoration has reduced ungulate browsing and facilitated recruitment of aspen (e.g., Painter et al. (2025); MacNulty et al. (2026); Painter et al. (2026) response). Although reductions in elk following wolf restoration have been linked to increased aspen recruitment, these discussions have primarily emphasized elk as the principal browser, although they also note increasing bison numbers and their potential impacts. Our findings support and extend this perspective by showing that browsing on riparian willows involves a broader herbivore assemblage, with bison exerting a substantial impact. Assessments of trophic cascade strength that focus primarily on elk may therefore overlook shifts in herbivore community composition and potential compensatory browsing by other ungulates. At the same time, our study is limited to riparian willows and does not directly address browsing dynamics in upland aspen stands, which have been central to the recent debate. Willow and aspen differ in growth form and habitat context, and their responses to changing herbivore communities may not be identical.

## Author Contributions

**Julia L. Jansson:** conceptualization (lead), data curation (equal), formal analysis (supporting), investigation (lead), methodology (equal), project administration (supporting), visualization (supporting), writing – original draft (supporting), writing – review and editing (equal). **Robert Spitzer:** conceptualization (supporting), data curation (equal), formal analysis (lead), methodology (equal), visualization (lead), writing – original draft (lead), writing – review and editing (equal). **Jaelle Caitlin Brealey:** formal analysis (equal), writing – review and editing (equal). **Göran Spong:** conceptualization (equal), formal analysis (supporting), funding acquisition (lead), methodology (equal), project administration (lead), writing – original draft (supporting), writing – review and editing (equal).

## Funding

This work was supported by Skogssällskapet and the “Gunnar and Lillian Nicholson Graduate Fellowship and Faculty Exchange Fund in Forestry” from NC State University (USA).

## Conflicts of Interest

The authors declare no conflicts of interest.

## Data Availability

The data supporting the findings of this study are publicly available in the Dryad repository at https://doi.org/10.5061/dryad.gtht76j1w.

## Appendix 1

**TABLE A1:** Study sites within Yellowstone National Park with geographic coordinates, elevation, and the number of successfully amplified browsing bite samples for six browsing species (moose *Alces alces* , North American *bison Bison bison* , elk *Cervus canadensis* , hare *Lepus townsendii* , deer *Odocoileus* sp., and bighorn sheep *Ovis canadensis* ). Site elevations (m a.s.l.) were extracted using the R package elevatr (Hollister et al. 2023), which retrieves digital elevation data from the openly available AWS Terrain Tiles (https://registry.opendata.aws/terrain‐tiles).

Site name	Lat (°)	Lon (°)	Elevation (m)	Number of amplified browsing bite samples						Total
				Moose	Bison	Elk	Hare	Deer	Bighorn sheep	
Blacktail Beaver	44.963917	−110.592150	1975	0	11	3	0	2	0	16
Crystal Creek	44.910935	−110.322070	1895	1	20	0	0	3	1	25
East Blacktail 2	44.947727	−110.563933	2072	4	1	1	1	5	1	13
Elk Creek EC	44.930702	−110.439705	1926	0	14	1	2	3	0	20
Elk Creek O	44.928453	−110.443731	1949	0	4	0	0	2	0	6
West Blacktail C	44.939557	−110.577088	2070	5	1	1	2	2	3	14

**TABLE A2:** Reference sequences used in custom Yellowstone mammalian database for DNA metabarcoding. Sequences were downloaded from Genbank.

Genbank accession	Position	Order	Family	Genus	Species/subspecies	Location of specimen	Length of sequence
M55540.1	1093–2665	Artiodactyla	Antilocapridae	Antilocapra	*Antilocapra americana*	Probably USA	1572
DQ318383.1		Artiodactyla	Bovidae	Bison	*Bison bison*	USA	1033
AY236429.1		Artiodactyla	Bovidae	Ovis	*Ovis aries*	Probably Italy	234
MK829158.1	1093–2666	Artiodactyla	Bovidae	Ovis	*Ovis aries*	China	1573
DQ318387.1		Artiodactyla	Bovidae	Ovis	*Ovis canadensis*	USA	1034
NC_015889.1	1093–2265	Artiodactyla	Bovidae	Ovis	*Ovis canadensis*	Canada	1172
JN315627.1		Artiodactyla	Cervidae	Alces	*Alces alces*	Canada	952
DQ318381.1		Artiodactyla	Cervidae	Cervus	*Cervus elaphus nelsoni* ^a^	Probably USA	1035
NC_007704.2	1094–2667	Artiodactyla	Cervidae	Cervus	*Cervus elaphus*	New Zealand	1573
DQ318366.1		Artiodactyla	Cervidae	Odocoileus	*Odocoileus hemionus*	Probably USA	1031
DQ318361.1		Artiodactyla	Cervidae	Odocoileus	*Odocoileus virginianus*	Probably USA	1031
JN632671.1	1091–2659	Artiodactyla	Cervidae	Odocoileus	*Odocoileus virginianus*	French Guiana	1568
KM612272.1	1092–2659	Artiodactyla	Cervidae	Odocoileus	*Odocoileus virginianus yucatanensis*	Mexico South SE	1567
M35874.1	1092–2658	Artiodactyla	Cervidae	Odocoileus	*Odocoileus virginianus*	USA	1566
DQ334833.1		Lagomorpha	Leporidae	Lepus	*Lepus americanus*	USA	1043
NC_024043.1	1092–2667	Lagomorpha	Leporidae	Lepus	*Lepus americanus*	USA	1575
NC_024041.1	1090–2665	Lagomorpha	Leporidae	Lepus	*Lepus townsendii*	USA	1575
FR691684.1	1104–2680	Rodentia	Castoridae	Castor	*Castor canadensis*	Finland	1576
KY321562.1	1104–2677	Rodentia	Castoridae	Castor	*Castor canadensis*	Canada	1573
NC_033912.1	1104–2677	Rodentia	Castoridae	Castor	*Castor canadensis*	Canada	1573
NC_042243.1	1111–2672	Rodentia	Sciuridae	Marmota	*Marmota flaviventris*	Probably Canada	1561
NC_080739.1	1108–2672	Rodentia	Sciuridae	Marmota	*Marmota monax*	Canada	1564
NC_048490.1	1109–2670	Rodentia	Sciuridae	Marmota	*Marmota vancouverensis*	Canada	1561
PX832459.1	1093–2675	Lagomorpha	Leporidae	Sylvilagus	*Sylvilagus nuttallii*	USA	1582
PQ664585.1	1092–2674	Lagomorpha	Leporidae	Sylvilagus	*Sylvilagus audubonii*	USA	1582
KP698976.1	1110–2676	Rodentia	Sciuridae	Urocitellus	*Urocitellus richardsonii*	Unknown	1566
DQ334842.1		Rodentia	Sciuridae	Tamiasciurus	*Tamiasciurus hudsonicus*	USA	1040
KY070171.1	1108–2690	Rodentia	Sciuridae	Tamias	*Tamias amoenus*	USA	1582
KY070152.1	1108–2691	Rodentia	Sciuridae	Tamias	*Tamias umbrinus*	USA	1583
AF147686.1		Rodentia	Sciuridae	Tamias	*Tamias minimus*	Canada	433
NC_005358.1	1100–2667	Lagomorpha	Ochotonidae	Ochotona	*Ochotona princeps*	Unknown	1567
NC_020630.1	1091–2664	Artiodactyla	Bovidae	Oreamnos	*Oreamnos americanus*	Probably France	1573
NC_091244.1	1114–2692	Perissodactyla	Equidae	Equus	*Equus caballus*	USA	1578
NC_003427.1	2235–3813	Carnivora	Ursidae	Ursus	*Ursus arctos*	Probably Canada	1578
NC_003426.1	2042–3621	Carnivora	Ursidae	Ursus	*Ursus americanus*	Probably Canada	1579
NC_012920.1	1671–3229	Primates	Hominidae	Homo	*Homo sapiens*	UK	1558
NC_010339.1	1094–2676	Rodentia	Muridae	Mus	*Mus musculus*	Unknown	1582
MZ901681.1	1455–3025	Artiodactyla	Bovidae	Bos	*Bos taurus*	Germany	1570

^a^ Until recently, red deer and elk were treated as a single species ( *Cervus elaphus* ) with multiple subspecies. Following taxonomic revision, American elk are now commonly recognized as *Cervus canadensis* , which we use throughout this paper, although some sources still list elk under *C. elaphus* or its subspecies.

**TABLE A3:** Summary of results from ASV taxonomic assignment. Target ASVs have been grouped by genus. Non‐target ASVs with good quality BLAST hits against the NCBI Genbank database have been grouped by genus for the most common taxa or by higher level taxonomic groups. For each of the three methods, the mean (and range) of the main metric used to assign high confidence or good quality assignments has been provided.

Target of study	BLAST Genbank quality	Taxa group	No. of ASVs	RDP genus‐level mean confidence score (range)	BLAST custom database mean % identity (range)	BLAST Genbank database mean % identity (range)
target	good quality	Genus: *Bison*	22	0.97 (0.9–1)	98.6 (97.8–100)	98.6 (97.8–100)
target	good quality	Genus: *Odocoileus*	15	0.97 (0.93–1)	98.6 (97.8–100)	98.6 (97.8–100)
target	good quality	Genus: *Alces*	12	0.99 (0.95–1)	98.6 (97.8–100)	98.6 (97.8–100)
target	good quality	Genus: *Ovis*	7	0.98 (0.95–1)	98.9 (97.8–100)	98.9 (97.8–100)
target	good quality	Genus: *Cervus*	8	0.98 (0.96–1)	98.8 (97.8–100)	98.8 (97.8–100)
target	good quality	Genus: *Lepus*	9	0.99 (0.98–0.99)	96.2 (94.6–96.8)	98.6 (97.8–98.9)
target	good quality	Family‐level or higher	8	0.72 (0.28–0.88)	93.5 (88.2–96.7)	96.8 (92.7–100)
non‐target^a^	good quality	Genus: *Homo*	53	1 (0.97–1)	98.7 (96.7–100)	99.2 (97.1–100)
non‐target^a^	good quality	Genus: *Mus*	17	0.99 (0.95–1)	95.4 (94.5–96.8)	98.8 (97.8–100)
non‐target^a^	good quality	Genus: *Bos*	6	0.96 (0.95–0.98)	98.2 (96.8–100)	98.7 (97.8–100)
non‐target	good quality	Genus: *Salix*	30	0.24 (0.01–0.62)	—	97.4 (94.6–100)
non‐target	good quality	Other mammals^b^	141	0.36 (0.11–0.9)	—	98.1 (88.9–100)
non‐target	good quality	Other eukaryotes	135	0.35 (0.07–0.81)	—	97.6 (84.4–100)
non‐target	good quality	Prokaryotes	48	0.33 (0.07–0.61)	—	95.1 (78–100)
non‐target	good quality	Viruses	115	0.49 (0.27–0.67)	—	96.5 (94–100)
non‐target	poor quality	NA	421	0.52 (0.06–0.99)	—	96.9 (85.7–100)
non‐target	no hits	NA	505	0.28 (0.04–0.73)	—	—

^a^ *Homo*, *Mus* and *Bos* reference sequences were included in the custom database, to identify potential contaminants.

^b^ ‘Other mammals’ includes the following genera (in order of decreasing frequency): *Sus*, *Canis*, *Vulpes*, *Felis*, *Capra*, *Lynx*, *Lycaon*, *Arvicola*, *Gulo*, *Talpa*, *Rucervus*, *Capreolus*, *Neomys*, *Pan* and *Rangifer*.

**TABLE A4:** Pairwise comparisons of browser community composition among study sites, on the basis of the proportional contribution of browsing bites by each species. Results are from chi‐square tests, with Holm‐adjusted *p*‐values shown. Bold values indicate significant Holm‐adjusted *p*‐values (*p* < 0.05). A significant value between two sites indicates a significant difference in the composition of browsing bite contributions among species. NA indicates cases where the test could not be performed because one or more browsing species were absent from both sites (i.e., the corresponding contingency table columns contained only zeros).

	Blacktail Beaver	Crystal Creek	East Blacktail 2	Elk Creek EC	Elk Creek O	West Blacktail C
Blacktail Beaver	—	NA	**0.006**	NA	NA	**0.001**
Crystal Creek		—	**0.002**	0.685	NA	**0.001**
East Blacktail 2			—	**0.006**	0.274	0.791
Elk Creek EC				—	NA	**0.004**
Elk Creek O					—	0.098
West Blacktail C						—
